# NKG2D ligand tumor expression and association with clinical outcome in early breast cancer patients: an observational study

**DOI:** 10.1186/1471-2407-12-24

**Published:** 2012-01-18

**Authors:** Esther M de Kruijf, Anita Sajet, Johanna GH van Nes, Hein Putter, Vincent THBM Smit, Robert A Eagle, Insiya Jafferji, John Trowsdale, Gerrit Jan Liefers, Cornelis JH van de Velde, Peter JK Kuppen

**Affiliations:** 1 Department of Surgery, Leiden University Medical Center, Leiden, the Netherlands; 2 Department of Medical Statistics, Leiden University Medical Center, Leiden, the Netherlands; 3 Department of Pathology, Leiden University Medical Center, Leiden, the Netherlands; 4 Cambridge Institute for Medical Research, University of Cambridge, Wellcome Trust/MRC Building, Addenbrookes Hospital, Hills Road, Cambridge, UK

**Keywords:** ULBP, MIC, NKG2D ligand, breast cancer, immune evasion

## Abstract

**Background:**

Cell surface NKG2D ligands (NKG2DL) bind to the activating NKG2D receptor present on NK cells and subsets of T cells, thus playing a role in initiating an immune response. We examined tumor expression and prognostic effect of NKG2DL in breast cancer patients.

**Methods:**

Our study population (n = 677) consisted of all breast cancer patients primarily treated with surgery in our center between 1985 and 1994. Formalin-fixed paraffin-embedded tumor tissue was immunohistochemically stained with antibodies directed against MIC-A/MIC-B (MIC-AB), ULBP-1, ULBP-2, ULBP-3, ULBP-4, and ULBP-5.

**Results:**

NKG2DL were frequently expressed by tumors (MIC-AB, 50% of the cases; ULBP-1, 90%; ULBP-2, 99%; ULBP-3, 100%; ULBP-4, 26%; ULBP-5, 90%) and often showed co-expression: MIC-AB and ULBP-4 (p = 0.043), ULBP-1 and ULBP-5 (p = 0.006), ULBP-4 and ULBP-5 (p < 0.001). MIC-AB (p = 0.001) and ULBP-2 (p = 0.006) expression resulted in a statistically significant longer relapse free period (RFP). Combined expression of these ligands showed to be an independent prognostic parameter for RFP (p < 0.001, HR 0.41). Combined expression of all ligands showed no associations with clinical outcome.

**Conclusions:**

We demonstrated for the first time that NKG2DL are frequently expressed and often co-expressed in breast cancer. Expression of MIC-AB and ULBP-2 resulted in a statistically significant beneficial outcome concerning RFP with high discriminative power. Combination of all NKG2DL showed no additive or interactive effect of ligands on each other, suggesting that similar and co-operative functioning of all NKG2DL can not be assumed. Our observations suggest that among driving forces in breast cancer outcome are immune activation on one site and tumor immune escape on the other site.

## Background

Breast cancer is the most commonly diagnosed female cancer and is the leading cause of death from cancer in women in the western world [1]. Decisions regarding use of systemic therapy are mainly based on prognostic and predictive factors like lymph node status, tumor size, grade, hormone receptor and human epidermal growth factor receptor 2 (HER2) expression [2,3]. However, current prognostic and predictive factors still do not provide optimal risk-stratification. Therefore, additional prognostic and predictive information could result in an improved tailored treatment for patients with breast cancer.

There is strong evidence that the immune system plays a role in tumor growth and progression [4,5]. An effective immune response may lead to recognition of tumor cells, resulting in their eradication. However, due to their genetic unstable nature, tumor cells may arise which display properties that enables them to escape from immune recognition [4,5]. Indeed, downregulation or loss of proteins that are crucial for immune responses, like classical human leukocyte antigens (HLA) class I, or upregulation of proteins that confer resistance to immune recognition, like non-classical HLA class I, are frequently found in various types of tumors [6-10].

The activating receptor natural killer cell lec-tin-like receptor gene 2D (NKG2D) is a stimulatory immune receptor that is expressed on natural killer (NK) cells, NKT cells, γδ^+ ^T cells and CD8+ T cells [11]. Ligands which bind NKG2D receptors comprise major histocompatibility complex class I chain-related proteins A and B (MIC-AB) and unique long 16 (UL16) binding proteins 1-6 (ULBP1-6) [12,13]. Expression of these ligands may be induced upon infection and other inducers of cellular stress and is unusual in normal cells [14]. By binding to the NKG2D receptors on NK and T cells, the NKG2D ligands may initiate an immune response against cells expressing these ligands. Overexpression and shedding of NKG2D ligands have been reported [14]. It is, however, unclear whether these features also results in activation of an immune response or lead to overstimulation and downregulation of NKG2D on immune cells [11].

Malignant transformation of cells may be among stimuli inducing expression of NKG2D ligands as such expression has been found in various tumor types [8-10,15-18]. This may be a mechanism for preventing tumor growth by advancing an anti-tumor immune response. Convincing evidence has been found in *in vivo *studies, which have shown that in mouse models transfection with NKG2D ligands resulted in a NKG2D-mediated tumor rejection [19,20]. Other studies showed that downregulation or complete knockout of NKG2D in mice resulted in an impaired immune response against tumor cells, higher expression levels of NKG2D ligands, and an increased incidence of certain tumors [21,22].

A few studies have investigated tumor expression of NKG2D ligands and associations with clinical outcome in human breast, colorectal, and ovarian cancer [8-10,15,16]. Expression of MIC-A was frequently found in all tumors studied and resulted in a statistically significant favorable patient's prognosis in colorectal cancer, while it was not statistically significantly associated with outcome in breast cancer and ovarian cancer [8-10,16]. ULBP1-5 expression was also found to be expressed in many tumor samples of colorectal and ovarian cancer [9,10,15]. In colorectal cancer expression of ULBP5 was an independent prognostic factor for a favorable clinical outcome [9]. In contrast to these results, expression of ULBP2 and ULBP4 were found to be independent prognostic factors for a worse outcome of ovarian cancer patients [10,15]. Taken together, several studies suggest that evasion of NKG2D-mediated immune regulation plays an important role in tumor progression, but some studies contradict this suggestion. Contradictory results may be explained by assuming functional differences in immune regulation of the different ligands. Moreover, expression of NKG2D ligands may behave different among different tumor types [9]. It is known that overexpression or shedding of these ligands leads to overstimulation and downregulation of NKG2D on immune cells [10,15], thereby evading an immune response.

In breast cancer, the prognostic effect of NKG2D ligands and their mutual relationship is largely unknown. Therefore, the purpose of this study was to analyze the clinical prognostic value of MIC-AB and ULBP1-5 in a large patient cohort of early stage breast cancer.

## Methods

### Patients and tumors

The patient population comprised all non-metastasized breast cancer patients primarily treated with surgery between 1985 and 1994 at the Leiden University Medical Center (n = 677). Patients with bilateral tumors or a prior history of cancer, other than basal cell carcinoma or cervical carcinoma *in situ*, were excluded. The following data were known: age, tumor morphology and differentiation grade, TNM stage, type of local and systemic therapy, recurrence and survival status, estrogen receptor (ER), progesterone receptor (PgR), and human epidermal growth factor 2 (HER2) expression (Table 1). All these parameters were determined according to current pathology standards. A tissue micro array (TMA) of available formalin-fixed paraffin-embedded (FFPE) tumors of the patient cohort has been previously constructed and described (n = 574) [23]. Approval was obtained from the Leiden University Medical Center Medical Ethics Committee. All samples were handled in a coded fashion, according to National ethical guidelines ("Code for Proper Secondary Use of Human Tissue", Dutch Federation of Medical Scientific Societies).

**Table 1:** Correlations between MIC-A-B, ULBP-1, ULBP-2 expression and well-established prognostic factors.

	Total		MICAB				ULBP1				ULBP2			
			
			Low		High		Low		High		Low		High	
	
	N	%	N	%	N	%	N	%	N	%	N	%	N	%
**Age**														
< 40 40-50 50-60 > = 60	48 145 132 249	8.4 25.3 23.0 43.4	4 28 26 42	4.0 28.0 26.0 42.0	31 86 78 163	8.7 24.0 21.8 45.5	22 62 45 91	10.0 28.2 20.5 41.4	10 32 44 79	6.1 19.4 26.7 47.9	34 85 100 172	8.7 21.7 25.6 44.0	9 42 20 52	7.3 34.1 16.3 42.3
**Grade**														
I II III	80 282 203	14.2 49.9 35.9	22 42 36	22.0 42.0 36.0	38 181 131	10.9 51.7 37.4	39 109 68	18.1 50.5 31.5	9 80 74	5.5 49.1 45.4	41 188 155	10.7 49.0 40.4	27 65 30	22.1 53.3 24.6
**Histological type**														
Ductal Lobular	513 53	90.6 9.4	91 9	91.0 9.0	322 29	91.7 8.3	194 22	89.8 10.2	151 12	92.6 7.4	354 30	92.2 7.8	106 16	86.9 13.1
**T-status**														
T1 T2 T3/4	211 272 72	38.0 49.0 13.0	40 44 12	41.7 45.8 12.5	124 176 47	35.7 50.7 13.5	96 87 31	44.9 40.7 14.5	37 96 26	23.3 60.4 16.4	128 198 54	33.7 52.1 14.2	59 46 12	50.4 39.3 10.3
**N-status**														
N0 N1-3	307 250	55.1 44.9	60 38	61.2 38.8	181 162	52.8 47.2	118 97	54.9 45.1	69 86	44.5 55.5	196 183	51.7 48.3	74 45	62.2 37.8
**ER-status**														
Negative Positive	203 337	37.6 62.4	33 65	33.7 66.3	137 212	39.3 60.7	95 122	43.8 56.2	55 103	34.8 65.2	147 238	38.2 61.8	45 75	37.5 62.5
**PgR-status**														
Negative Positive	223 313	41.6 58.4	33 66	33.3 66.7	147 198	42.6 57.4	88 127	40.9 59.1	68 90	43.0 57.0	169 217	43.8 56.2	40 78	33.9 66.1
**Her2-status**														
No overexpression- Overexpression	378 44	89.6 10.4	78 6	92.9 7.1	264 36	88.0 12.0	174 20	89.7 10.3	125 15	89.3 10.7	291 29	90.9 9.1	79 14	84.9 15.1
**Local Therapy**														
MAST-RT MAST+RT BCS-RT BCS+RT	223 108 5 238	38.9 18.8 0.9 41.5	41 17 0 42	41.0 17.0 0.0 42.0	146 66 5 141	40.8 18.4 1.4 39.4	80 46 2 92	36.4 20.9 0.9 41.8	79 33 2 51	47.9 20.0 1.2 30.9	149 83 5 154	38.1 21.2 1.3 39.4	53 15 0 55	43.1 12.2 0.0 44.7
**Systemic therapy**														
CT alone HT alone CT&HT None	112 75 18 369	19.5 13.1 3.1 64.3	17 8 1 74	17.0 8.0 1.0 74.0	73 54 13 218	20.4 15.1 3.6 60.9	44 31 3 142	20.0 14.1 1.4 64.5	25 29 9 102	15.2 17.6 5.5 61.8	80 54 14 243	20.5 13.8 3.6 62.1	24 16 3 80	19.5 13.0 2.4 65.0
**Total**	**574**	**100**	**100**	**100**	**358**	**100**	**220**	**100**	**165**	**100**	**391**	**100**	**123**	**100**

Missing values are not shown.

***Abbreviations ***N number of patients; % percentage; ER estrogen receptor; PgR progesterone receptor; HER2 human epidermal growth factor receptor 2; MAST mastectomy; RT radiotherapy; BCS breast conservative surgery; ET endocrine therapy; CT chemotherapy.

### Immunohistochemistry

Antibodies specific against MIC-AB (ab54413; Abcam), ULBP-1 (HPA007547; Atlas antibodies), ULBP-2 (af1298; R&D systems), ULBP-3 (CUMO3-100; BAMOMAB), ULBP-4 (RAET1E) and ULBP-5 (RAET1G, both kindly provided by Dr. Robert A Eagle, Cambridge, UK) [9,24] were used for immunohistochemical staining of tumor tissue. The specificity of anti-ULBP-2 antibody has been previously determined, which showed occassional cross-reactity with highly related molecules RAET1L and to a lesser extent with RAET1G, but a good recognition of ULBP-2 [25]. We are not aware of antibodies which can specifically discriminate between ULBP2, RAET1L and RAET1G extracellular domains.

TMA sections of 4 μm were cut, deparaffinized and rehydrated. Endogenous peroxidase was blocked in 0.3% hydrogen-peroxide methanol for 20 minutes. Heat-induced antigen retrieval for 10 minutes at maximum power in a microwave oven was performed. Sections were incubated overnight with primary antibodies using predetermined optimal dilutions and incubations times. Sections for ULBP-2 staining were incubated with Rabbit Anti-Goat Immunoglobulins (DAKO) followed by StreptABComplex (DAKO) for 30 minutes. Sections for all other stainings were incubated with secondary antibody Envision (Dako cytomation K4001 or K4003) for 30 minutes. Stainings were visualized using DAB-solution (Dako cytomation K3468), counterstained with haematoxylin, dehydrated, and finally mounted in malinol. For each type of antibody, all tissue sections were stained simultaneously to avoid inter-assay variation.

### Evaluation of immunostaining

Microscopic analysis of MIC-AB, ULBP-1, ULBP-2, ULBP-3, ULBP-4 and ULBP-5 expression was performed by two independent observers in a blinded manner. Since staining of tumors was relatively homogenous, for each tumor the overall intensity of staining (negative (0), weak (1), intermediate (2) or strong (3)) was determined.

### Statistical analysis

Statistical analyses were performed using the statistical package SPSS (version 16.0 for Windows, Spps Inc, Chicago, IL, USA). Cohen's kappa coefficient was used to assess inter-observer agreement in quantification. This revealed a moderate agreement for ULBP-5 (kappa = 0.410), a substantial agreement in classification for MIC-AB (kappa = 0.790) and ULBP-4 (kappa = 0.650), and an almost perfect agreement for ULBP-1 (kappa = 0.913), ULBP-2 (kappa = 0.940), and ULBP-3 (kappa = 0.869). The χ^2 ^test was used to evaluate associations between expression of the different NKG2D ligands. Relapse free period (RFP) was the time from date of surgery until an event (locoregional recurrence and/or a distance recurrence, whichever came first). The Kaplan-Meier method was used for survival plotting and log-rank test for comparison of survival curves. RFP is reported as cumulative incidence function, after accounting for death as competing risk [26]. Cox regression was used for univariate and multivariate analysis for RFP. Significant variables (p < 0.1) in univariate analysis were included in multivariate analysis.

## Results

### Patient and tumor characteristics

Median age of patients was 57 years (range: 23-96 years). Median follow-up of patients alive was 19 years (range: 14-23 years). Clinicopathological and treatment characteristics are shown in table 1.

### Expression of NKG2D ligands

**Figure 1:**
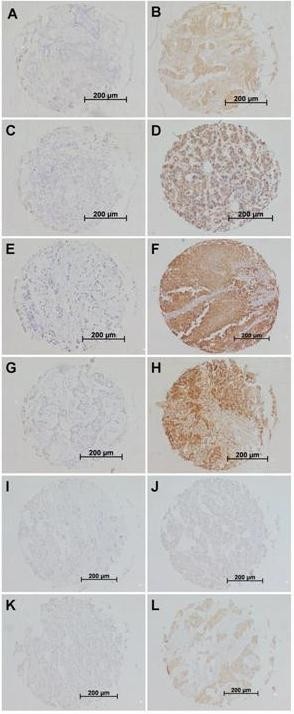
**Representative examples of immunohistochemical stainings of primary breast cancer tissues for respectively no expression and high expression of MIC-AB (A: intensity 0 (negative); B: intensity 2 (intermediate)), ULBP-1 (C: intensity 0 (negative); D: intensity 2 (intermediate)), ULBP-2 (E: intensity 0 (negative); F: intensity 3 (strong)), ULBP-3 (G: intensity 0 (negative); H: intensity 3 (strong)), ULBP-4 (I: intensity 0 (negative); J: intensity 1 (weak)), and ULBP-5 (K: intensity 0 (negative); L: intensity 3 (strong)) in breast cancer**. Immunohistochemistry was performed according to standard protocols as described in Materials and Methods.

Most of the NKG2D ligands examined in this study were frequently expressed among the breast tumor cohort: MIC-AB in 50% of the cases; ULBP-1 in 90%; ULBP-2 in 99%; ULBP-3 in 100%; ULBP-4 in 26%; and ULBP-5 in 90%. A broad distribution of immunohistochemical staining-intensities was seen for ULBP-2, ULBP-3 and ULBP-5, while MIC-AB, ULBP-1 and ULBP-4 showed a skewed distribution of staining-intensities where most tumors stained weakly positive (representative examples of staining: Figure 1). Therefore, the median intensity was taken as a cut-off value for all ligands to categorize low and high expression resulting in respectively 50%, 43%, 24%, 27%, 26%, 10% of tumors with high expression of MIC-AB (Figure 1B), ULBP-1 (Figure 1D), ULBP-2 (Figure 1F), ULBP-3 (Figure 1H), ULBP-4 (Figure 1J) and ULBP-5 (Figure 1L) and respectively 50%, 57%, 76%, 73%, 90% of the tumors with low expression of MIC-AB (Figure 1A), ULBP-1 (Figure 1C), ULBP-2 (Figure 1E), ULBP-3 (Figure 1G), ULBP-4 (Figure 1I) and ULBP-5 (Figure 1K).

NKG2D ligands were found to be frequently co-expressed: MIC-AB positively correlated with ULBP-4 (p = 0.043); ULBP-1 showed a positive correlation with ULBP-5 (p = 0.006); ULBP-4 had a positive correlation with ULBP-5 (p < 0.001).

### Association of NKG2D ligands with clinicopathological parameters

High expression of NKG2D ligands was generally associated with favorable clinicopathological parameters (table 1 and 2): statistically significant associations were found between high expression of MIC-AB and lower tumor grade (p = 0.012); high expression of ULBP-1 and higher tumor grade (p < 0.001), smaller tumor size (p < 0.001) and more lymph node positive tumors (p = 0.049); high expression of ULBP-2 and younger age (p = 0.022), lower tumor grade (p < 0.001), smaller tumor size (p = 0.005) and more lymph node negative tumors (p = 0.046); high expression of ULBP-3 and higher tumor grade (p = 0.001); high expression of ULBP-4 and smaller tumor size (p = 0.001); high expression of ULBP-5 and more PgR negative tumor status (p = 0.016).

**Table 2:** Correlations between ULBP-3, ULBP-4 and ULBP-5 expression and well-established prognostic factors.

	Total		ULBP3				ULBP4						ULBP5	
			
			Low		High		Low		High		Low		High	
	
	N	%	N	%	N	%	N	%	N	%	N	%	N	%
**Age**														
< 40 40-50 50-60 > = 60	48 145 132 249	8.4 25.3 23.0 43.4	25 84 68 128	8.2 27.5 22.3 42.0	11 24 22 55	9.8 21.4 19.6 49.1	29 91 86 164	7.8 24.6 23.2 44.3	15 33 30 49	11.8 26.0 23.6 38.6	38 119 103 185	8.5 26.7 23.1 41.6	3 9 12 23	6.4 19.1 25.5 48.9
**Grade**														
I II III	80 282 203	14.2 49.9 35.9	45 160 96	15.0 53.2 31.9	10 43 57	9.1 39.1 51.8	51 181 134	13.9 49.5 36.6	13 67 43	10.6 54.5 35.0	62 219 157	14.2 50.0 35.8	2 21 23	4.3 45.7 50.0
**Histological type**														
Ductal Lobular	513 53	90.6 9.4	281 20	93.4 6.6	102 8	92.7 7.3	330 37	89.9 10.1	113 10	91.9 8.1	396 43	90.2 9.8	43 3	93.5 6.5
**T-status**														
T1 T2 T3/4	211 272 72	38.0 49.0 13.0	113 142 39	38.4 48.3 13.3	40 54 17	36.0 48.6 15.3	118 180 60	33.0 50.3 16.8	57 61 6	46.0 49.2 4.8	159 209 64	36.8 48.4 14.8	19 26 2	40.4 55.3 4.3
**N-status**														
N0 N1-3	307 250	55.1 44.9	158 135	53.9 46.1	61 49	55.5 44.5	193 170	53.2 46.8	66 55	54.5 45.5	237 196	54.7 45.3	24 21	53.3 46.7
**ER-status**														
Negative Positive	203 337	37.6 62.4	113 178	38.8 61.2	37 73	33.6 66.4	135 222	37.8 62.2	43 80	35.0 65.0	152 278	35.3 64.7	21 25	45.7 54.3
**PgR-status**														
Negative Positive	223 313	41.6 58.4	130 159	45.0 55.0	42 68	38.2 61.8	141 215	39.6 60.4	51 72	41.5 58.5	158 267	37.2 62.8	26 21	55.3 44.7
**Her2-status**														
Overexpression - Overexpression +	378 44	80.9 19.1	207 24	89.6 10.4	82 10	89.1 10.9	256 33	88.6 11.4	93 6	93.9 6.1	311 32	90.7 9.3	35 6	85.4 14.6
**Local Therapy**														
MAST-RT MAST+RT BCS-RT BCS+RT	223 108 5 238	38.9 18.8 0.9 41.5	116 56 3 130	38.0 18.4 1.0 42.6	43 24 1 44	38.4 21.4 0.9 39.3	141 86 1 142	38.1 23.2 0.3 38.4	46 14 3 64	36.2 11.0 2.4 50.4	165 94 4 182	37.1 21.1 0.9 40.9	24 3 0 20	51.1 6.4 0.0 42.6
**Systemic therapy**														
CT alone HT alone CT&HT None	112 75 18 369	19.5 13.1 3.1 64.3	65 32 10 198	21.3 10.5 3.3 64.9	23 10 2 77	20.5 8.9 1.8 68.8	81 52 13 224	21.9 14.1 3.5 60.5	20 16 1 90	20.3 12.6 0.8 70.9	93 60 11 281	20.9 13.5 2.5 63.1	8 5 1 33	17.0 10.6 2.1 70.2
**Total**	**574**	**100**	**305**	**100**	**112**	**100**	**370**	**100**	**127**	**100**	**445**	**100**	**47**	**100**

Missing values are not shown.

***Abbreviations ***N number of patients; % percentage; ER estrogen receptor; PgR progesterone receptor; HER2 human epidermal growth factor receptor 2; MAST mastectomy; RT radiotherapy; BCS breast conservative surgery; ET endocrine therapy; CT chemotherapy.

### Associations with outcome of NKG2D ligands

**Figure 2:**
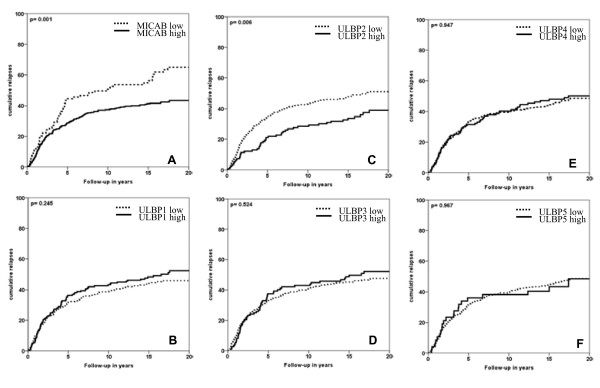
**Relapses over time related with expression of MIC-AB (A), ULBP-1 (B), ULBP-2 (C), ULBP-3 (D), ULBP-4 (E), and ULBP-5 (F)**. X-axis represents patient follow-up in years; Y-axis represents cumulative relapses in %. Log-rank p-values are shown in each graph. Only expression of MIC-AB and ULBP-2 resulted in statistically significantly favorable relapse-free period (RFP).

When analyzed separately, MIC-AB and ULBP-2 showed statistically significant results on outcome analyses (log rank p-values respectively: 0.001, 0.006), where high expression of MIC-AB and ULBP-2 showed to have fewer relapses over time compared to low expression (Figure 2A, C). For MIC-AB low expression, 51% of patients were relapse free after 20 years, while of patients with high expression of MIC-AB 27% showed a relapse within 20 years. For ULBP-2, 20 year RFP rates for low expression versus high expression were respectively 56% and 43%. No statistically significant associations with outcome were seen for ULBP-1, ULBP-3, ULBP-4 and ULBP-5 (Figure 2B, D-F).

Cox univariate regression analysis was performed for expression of each type of ligand. MIC-AB (Hazard ratio (HR) 0.60, 95% confidence interval (95%CI) 0.448-0.810, p = 0.001) and ULBP-2 (HR 0.63, 95%CI 0.454-0.869, p = 0.005) showed statistically significant results for a favorable RFP, while all other types of ligands did not reach statistical significance (data not shown).

**Figure 3:**
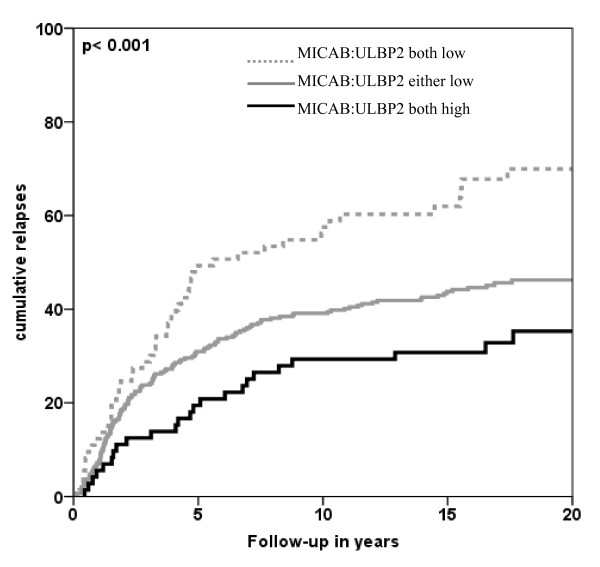
**Relapses over time related with combined expression of MIC-AB and ULBP-2**. X-axis represents patient follow-up in years; Y-axis represents cumulative relapses in %. Log-rank p-values are shown in the graph. Combined low expression of MIC-AB and ULBP-2 resulted in the worst outcome of patients concerning relapse-free period (RFP); while combined high expression of both ligands resulted in the most favorable outcome of patients.

To seek how combined expression of MIC-AB and ULBP2 ligands would predict patient outcome a new variable was made representing expression of both ligands: (1) Both MIC-AB and ULBP-2 low expression; (2) either MIC-AB or ULBP-2 high expression; (3) both MIC-AB and ULBP-2 high expression. Combined expression of MIC-AB and ULBP-2 resulted in a prognostic factor (log rank p-value: < 0.001; Figure 3), where low expression of both ligands versus high expression of either ligand versus high expression of both ligands resulted in respectively 23%, 48% and 60% of patients to be relapse free after 20 years. Cox proportional multivariate analysis showed the combined ligand variable to be statistically significant for RFP independently of known clinicopathological parameters (MIC-AB and ULBP-2 both low versus either high: HR 0.54, 95%CI 0.380-0.757; MIC-AB and ULBP-2 both low versus both high: HR 0.41, 95%CI 0.246-0.682; p-value < 0.001) (Table 3).

**Table 3:** Cox univariate and multivariable analysis for recurrence free period (RFP) for combined expression of MIC-AB and ULBP-2.

Relapse Free Period		UNIVARIATE			MULTIVARIATE		
	
	N	HR	95% CI	p-value	HR	95% CI	p-value
**Age**							

< 40	48	1.00		0.422			
40-50	145	0.97	0.612-1.539				
50-60	132	1.17	0.734-1.853				
> 60	249	0.90	0.574-1.408				

**Grade**							

I	80	1.00		0.001	1.00		0.473
II	282	1.43	0.945-2.172		1.18	0.711-1.948	
III	203	2.02	1.326-3.078		1.34	0.802-2.231	

**Histological type**							

Ductal	513	1.00		0.291			
Other	53	1.24	0.832-1.846				

**Tumor stage**							

pT1	211	1.00		< 0.001	1.00		0.298
pT2	272	1.59	1.205-2.093		1.17	0.832-1.637	
pT3/4	72	2.49	1.706-3.635		1.45	0.908-2.316	

**Nodalstage**							

pN-	307	1.00		< 0.001	1.00		< 0.001
pN+	250	3.06	2.379-3.945		2.70	1.987-3.669	

**ER-status**							

Negative	203	1.00		0.725			
Positive	337	1.05	0.808-1.359				

**PgR-status**							

Negative	223	1.00		0.744			
Positive	313	0.96	0.743-1.236				

**HER2**							

No overexpression	378	1.00		0.401			
Overexpression	44	1.21	0.776-1.883				

**Endocrine therapy**							

ET-	481	1.00		0.197			
ET+	93	1.24	0.896-1.705				

**Chemotherapy**							

CT-	444	1.00		0.839			
CT+	130	0.97	0.730-1.291				

**MIC-AB & ULBP-2**							

Both Low	68	1.00		< 0.001	1.00		< 0.001
Either one high	275	0.59	0.426-0.820		0.54	0.380-0.757	
Both high	64	0.38	0.230-0.612		0.41	0.246-0.682	

Missing values are not shown.

***Abbreviations ***N number of patients; HR hazard ratio; 95%CI 95% Confidence Interval; ER estrogen receptor; PgR progesterone receptor; HER2 human epidermal growth factor receptor 2; ET endocrine therapy; CT chemotherapy.

In order to analyze the frequencies and prognostic effect of number of co-expressed and amount of co-expression of NKG2D ligands, two new variables were constructed. First, the total number of the different NKG2D ligands that were expressed. For that purpose, the number of NKG2D ligands with high expression was counted. So for each tumor, this resulted in a minimal and maximal possible score of respectively 0 and 6. Second, the total amount of NKG2D ligand expression. For that purpose, the intensity of staining (ranging from 0 to 3) of NKG2D ligands was added, obtaining a total NKG2D ligand intensity score. So for each tumor, this resulted in a minimal and maximal possible score of respectively 0 and 18.

The median number of NKG2D ligands with high expression was 1 (range 0-6). For statistical reasons (too small patient groups) in outcome analyses, the groups with 3, 4, 5 and 6 numbers of different NKG2D ligands highly expressed were combined as one single group: ≥ 3 ligands of high expression.

**Figure 4:**
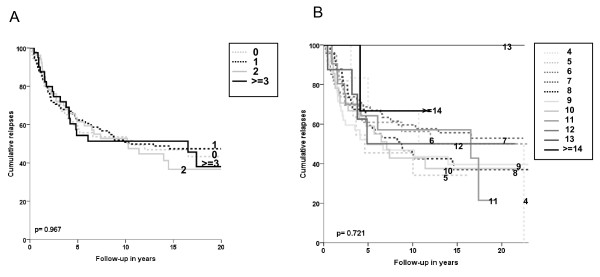
**Relapses over time related with combined number of NKG2D ligands with high expression (A) and amount of expression of NKG2D ligands (B)**. (A) legends in graph show total number of NKG2D ligands with high expression; (B) legends in graph show total intensity score of all NKG2D ligand expression. X-axis represents patient follow-up in years; Y-axis represents cumulative relapses in %. Log-rank p-values are shown in the graph. No associations were found with outcome concerning RFP for either combined number of expressed (A) or combined amount of expression (B) of ligands.

No associations were seen for the number of NKG2D ligands with high expression for RFP outcome analyses (log rank p-value: 0.967); patients with tumors with a low number of NKG2D ligands with high expression resulted in a similar RFP compared to a high number of NKG2D ligands with high expression (Figure 4A).

The median total amount of NKG2D ligand intensity score was 8 (range 4-16). No tumors showed complete lack (score 0) or high intensity expression of all NKG2D ligands (score 18).

For outcome analyses, NKG2D scores 14-16 were combined and classified as ≥ 14, since these subgroups separately contained only one patient. No association was seen for amount of total NKG2D ligand expression and RFP (log rank p-value: 0.721); high total NKG2D ligand expression resulted in some cases in a worse RFP (e.g. score 11) while in others it resulted in a favorable RFP (e.g. score 13) and vice versa, low total NKG2D ligand expression resulted for some patients in a favorable RFP (e.g. score 6) and for other patients in a worse RFP (e.g. score 4) (Figure 4B).

## Discussion

The importance of interaction between tumor development and the immune system for cancer outcome is highlighted by an overwhelming number of studies, performed *in vitro, in vivo *and using patient cohorts. Recent studies have shown that NKG2D ligands may play an important role in cancer immunosurveillance and cancer immunoediting [8-10,15-22]. In this study, we examined the impact of tumor expression of NKG2D ligands on the prognosis of breast cancer patients. The data of our study indicate that NKG2D ligands are frequently high expressed in breast tumors and that this expression influences prognosis of patients. We were able to statistically prove that high expression levels of MIC-AB and ULBP-2 resulted in a RFP benefit. Combining expression of MIC-AB and ULBP-2 resulted in a very accurate stratification of patients for prognosis concerning RFP. The prognostic potential of this combined variable was comparable to that of lymph node status: patients with low tumor expression of both ligands had an almost 2.5 times increased risk of developing relapses compared to patients with high tumor expression of both ligands.

NKG2D ligands are expressed on the cell surface in response to stress or malignant transformation [11]. Our study confirms that breast cancer tumor cells show frequent and high expression of NKG2D ligands, as has been found in other studies for various types of tumors such as ovarian cancer, colorectal cancer and breast cancer [8-10,15,16]. Though all studies show consistently frequent expression of NKG2D ligands, very diverse prognostic effects have been described for these types of ligands in different cancer types [8-10,15,16]. This may be explained by functional differences in immune regulation for varying expression levels of different ligands in different environments. Expression of NKG2D ligands may induce an immune response through binding to the NKG2D receptor, present on NK cells and a subset of T cells [11]. Therefore, selective outgrowth of malignant cells that do not express these NKG2D ligands may be a mechanism of tumor immune escape. On the other hand, overexpression of NKG2D ligands could lead to overstimulation and thereby insensibility or anergy of immune cells, which would result in evasion of immune attack by tumors overexpressing NKG2D ligands [11]. Adding to this hypothesis, it has been reported that NKG2D ligands on the cell membrane may be cleaved and produce soluble molecules. This shedding of NKG2D ligands could systemically downregulate NKG2D receptor expression and thereby result in an impaired anti-tumor reactivity of NK and T cells [11,27]. Taken together, the mechanisms by which NKG2D ligands mediate immune function or dysfunction may be diverse in different tumors and differ according to circumstances. The contradictory results on the prognostic effect of NKG2D ligands found between different studies on different tumors may be reflected by the functional and mechanistic implications of interaction between NKG2D and its ligands. In ovarian cancer expression of NKG2D ligands resulted in a worse patient outcome, probably due to chronic overexpression and shedding of these ligands, leading to overstimulation and downregulation of the NKG2D receptor of NK and T cells and, therefore, an impaired immune response [10,15]. Supporting the hypothesis that elevated expression of NKG2D ligands results in immune escape in ovarian cancer, one study found elevated levels of MIC-AB and ULBP-2 to be positively correlated to less intra-tumor epithelial CD57+ cells. The results found in breast cancer in the present study are contradictory to the results found in ovarian cancer, but similar to those found in colorectal cancer [9,16]. The results in our study and colorectal cancer are supported by the theory that expression of NKG2D ligands results in activation of immune cells which is reflected in a patient beneficial outcome for high ligand expression [9,16]. We found frequent and high expression of ligands in our study and statistically significant associations between expression levels of these ligands, indicating their cooperation with each other. Adding to the hypothesis that low expression of these ligands is a result of selective pressure by the immune system that results in cancer immune evasion or immunoediting, low expression of MIC-AB and ULBP-2 were prognostic factors for an unfavorable RFP of patients. When expression of MIC-AB and ULBP-2 were combined they showed to add to each others prognostic effect which is in line with the results found in previous studies [9,10] and suggests that NKG2D ligands operate together and in a similar manner.

Since the exact functioning of all NKG2D ligands and their cooperative function is largely unknown, we performed outcome analyses with two different variables that represented combined number of highly co-expressed ligands and amount of co-expression of all ligands. The results of these analyses revealed no patterns of any cooperative functioning between all ligands, as both variables showed no consistent and significant relationship with clinical outcome of disease. This suggests that the original hypothesis of all NKG2D ligands having a similar functioning and additive effect on each other's functioning in activating or evading an immune response, may be too simplistic. Considering our results and those as found in literature, altogether, each NKG2D ligand analysed separately does not show equal effects on clinical outcome, and different ligands show varying prognostic effects in different tumors. Specific combinations of ligands (e.g. MIC-AB and ULBP-2 in our study, ULBP2 and ULBP4 in ovarian cancer [10]) do show additive effects or statistical interactions on prognostic value. However, as highlighted by our combined analyses, a simple additive effect of all NKG2D ligands, by considering a similar or cooperative functioning of all these ligands, can not be assumed. This indicates the complexity of NKG2D ligands functioning and emphasizes the importance of further research on the precise mechanisms of actions of NKG2D ligands, separately, in combination with each other, and under different circumstances.

## Conclusions

We have shown in this study, for the first time, that breast tumors may express all of the known NKG2D ligands and that expression of MIC-AB and ULBP-2 results in a favorable outcome concerning RFP. A variable combining MIC-AB and ULBP-2 expression has shown to be a prognostic parameter independently of known clinicopathological parameters and with high discriminative power. Our results suggest that NKG2D ligands play a crucial role in tumor immunoediting in breast cancer and provide further evidence that tumor-immune interactions play an important role in breast cancer. In addition, by NKG2D ligand combined analyses we highlight the importance of further studies on unraveling the precise separate functioning of these ligands.

## List of abbreviations

%: percentage; 95%CI: 95 percent confidence interval; BCS: breast conservative surgery; CT: chemotherapy; ER: estrogen receptor; ET: endocrine therapy; FFPE: formalin-fixed paraffin-embedded; HER2: human epidermal growth factor receptor 2; HLA: human leukocyte antigen; HR: hazard ratio; MAST: mastectomy; MIC-AB: major histocompatibility complex class I chain-related proteins A and B; N: number; NK cell: natural killer cell; NKG2D: natural killer cell lec-tin-like receptor gene 2D; NKT cell: natural killer T cell; PgR: progesterone receptor; RFP: relapse free period; RT: radiotherapy; TMA: tissue micro array; UL16: unique long 16; ULBP: unique long 16 binding protein

## Competing interests

The authors declare that they have no competing interests.

## Authors' contributions

EdK participated in the design of the study, the collection of tumor material, the construction of the tissue micro array, the immunohistochemical stainings, quantification and acquisition of data, performed the statistical analysis and participated in writing of the manuscript. AS contributed in the collection of tumor material, immunohistochemical stainings and quantifications. JvN contributed in the collection of tumor material, acquisition of data and writing of the manuscript. HP carried out statistical analysis and contributed in the writing of the manuscript. VS contributed in the collection of tumor material, quantification and acquisition of data and writing of the manuscript. RE created, tested and provided the antibodies and contributed in acquisition of data and writing of the manuscript. IJ created, tested and provided the antibodies and contributed in acquisition of. JT created, tested and provided the antibodies and contributed in acquisition of data. GJL participated in the design of the study and coordination and helped to draft the manuscript. CvdV participated in the design of the study and coordination and helped to draft the manuscript. PK participated in the design and coordination of the study and writing of the manuscript coordination and helped to draft the manuscript. All authors read and approved the final manuscript.

## Pre-publication history

The pre-publication history for this paper can be accessed here:

http://www.biomedcentral.com/1471-2407/12/24/prepub
